# Genome *In Silico* and *In Vitro* Analysis of the Probiotic Properties of a Bacterial Endophyte, *Bacillus Paranthracis* Strain MHSD3

**DOI:** 10.3389/fgene.2021.672149

**Published:** 2021-11-11

**Authors:** Mamonokane Olga Diale, Eugenie Kayitesi, Mahloro Hope Serepa-Dlamini

**Affiliations:** ^1^ Department of Biotechnology and Food Technology, University of Johannesburg, Johannesburg, South Africa; ^2^ Department of Consumer and Food Science, University of Pretoria, Pretoria, South Africa

**Keywords:** *Bacillus* paranthracis, probiotics, biosynthetic gene clusters, endophytes, secondary metabolites

## Abstract

Spore*-*forming *Bacillus* species are gaining interest in human health recently, due to their ability to withstand the harsh environment of the gastrointestinal tract. The present study explores probiotic features of *Bacillus paranthracis* strain MHSD3 through genomic analysis and *in vitro* probiotic assays. The draft genome of strain MHSD3 contained genes associated with tolerance to gastrointestinal stress and adhesion. Cluster genes responsible for the synthesis of antimicrobial non-ribosomal peptide synthetases, bacteriocins, and linear azole-containing peptides were identified. Additionally, strain MHSD3 was able to survive in an acidic environment, had the tolerance to bile salt, and exhibited the capability to tolerate gastric juices. Moreover, the isolate was found to possess strong cell surface traits such as high auto-aggregation and hydrophobicity indices of 79 and 54%, respectively. Gas chromatography–mass spectrometry analysis showed that the strain produced secondary metabolites such as amino acids, phenolic compounds, and organic acid, known to exert health-promoting properties, including the improvement of gastrointestinal tract health.

## Introduction

Probiotics have gained attention globally as an alternative to alleviate or prevent gastrointestinal diseases such as diarrheal diseases, and inflammatory bowel diseases (Cui et al., 2020). Most studies on probiotic microorganisms are mainly on traditionally known probiotic groups such *Lactobacillus*, *Bifidobacteria*, *Propionibacterium*, *Streptococcus*, and some *Saccharomyces* species. Although these species show outstanding probiotic properties, some *Lactobacillus* and *Bifidobacteria* species perform poorly in acidic environments (Ruiz et al., 2011; Cao et al., 2020). Thus, new species are sought as probiotics, of which *Bacillus* species as probiotics have recently gained interest, as these are able to produce spores that allow for survival in harsh environments (Elisashvili et al., 2019). Spore-forming bacteria offer an advantage over common probiotic species, as they are able to survive in extremely harsh environments and sustain stability during heat processing and low-temperature storage (Lefevre et al., 2017). Additionally, *Bacillus* species are known to produce a broad spectrum of secondary metabolites, which include antibiotics and lipopeptides such as iturin, surfactin, fengycins, and bacteriocins, which have antimicrobial activity (Susic et al., 2020). *Bacillus* species are a good source of antimicrobial peptides (Sumi et al., 2015). This is compounded with vitamins, amino acids, antioxidants, and immuno-modulatory compounds, which makes *Bacillus* species better probiotic species (Sansinenea and Ortiz, 2011; Kuebutornye et al., 2019). The production of antimicrobial compounds is one of the mechanisms by which probiotic bacteria exert an advantageous effect on the host, presumably by eliminating the growth and colonization of gastrointestinal tract pathogenic bacteria (Vieco-Saiz et al., 2019).

*Bacillus paranthracis* strain MHSD3 is a bacterial endophyte isolated from the medicinal plant *Pellaea calomelanos* and was initially identified as a *Bacillus infantis* strain by phylogenetic analysis of its partial 16S ribosomal RNA gene (Mahlangu and Serepa-Dlamini, 2018). Endophytes are endosymbiotic microorganisms such as bacteria and fungi that colonize the internal tissues of plants without causing any infection (Khan et al., 2020). Nearly 300,000 plant species exist on earth, and it is estimated that each plant hosts one or more endophytes (Strobel et al., 2004). Endophytes are beneficial to the plant hosts by promoting plant growth (Santos et al., 2018), increase nutrient uptake (da Silva Ribeiro et al., 2018), play a role as bio-fertilizers and biocontrol agents (Selim et al., 2012; Santos et al., 2018), and produce beneficial secondary metabolites (Brader et al., 2014; Palanichamy et al., 2018). The plant, in turn, provides an environment for survival and carbon for the endophytes. Bacterial endophytes are equipped with arrays of secondary metabolites, but their metabolic potential is less investigated (Brader et al., 2014). Endophytic bacteria are reported for producing various secondary metabolite classes such as alkaloids, polyketones, steroids, flavonoids, terpenoids, peptides, quinols, and phenols (Singh et al., 2017). These metabolites are of importance in agricultural, pharmaceutical, and industrial fields and are used for the development of antibiotics, anticancer, antifungal, antiviral, insecticidal, and immunosuppressant compounds (Singh et al., 2017; Strobel, 2018). Bacterial endophytes have gained interest as a source of bioactive compounds utilized in various industries such as pharmaceutical, agriculture, and food (Aswani et al., 2020). In this study, *B. paranthracis* strain MHSD3, a bacterial endophyte isolated from *P. calomelanos*, was sequenced to unveil the genetic basis of its safety and probiotic ability; its bioactive secondary metabolites were identified using gas chromatography–mass spectrometry. In addition, the *in vitro* probiotic potential of strain MHSD3 was investigated.

## Materials and Methods

### Isolation of the Bacterial Strain

*B. paranthracis* strain MHSD3 was isolated from sterilized leaves of the medicinal plant *P. calomelanos*, as described by Mahlangu and Serepa-Dlamini (2018). It was initially identified by sequencing the partial 16S ribosomal RNA gene (GenBank accession number: MF613649). Thirty percent glycerol stocks of the bacterial culture were plated on nutrient agar (NA) plates and incubated for 24–48 h at 28°C; for routine culture maintenance, the bacteria were grown on nutrient broth (NB) at 28°C and preserved in 30% glycerol (v/v) at -80°C for long-term storage.

### Genomic Deoxyribonucleic Acid Isolation, Library Preparation, and Sequencing

The genomic DNA was extracted from solid colonies using a NucleoSpin microbial DNA extraction kit following the manufacturer’s protocol (Macherey-Nagel, Germany). The DNA was sequenced at a commercial service provider, Biotechnology Platform, Agricultural Research Council, Onderstepoort, South Africa. Paired-end libraries (2 × 150  bp) were generated using the NextEra DNA sample preparation kit (Illumina, United States), and sequencing was performed on the HiSeq 2,500 platform.

### Genome Assembly and Annotation

The quality control, trimming, and assembly were performed on GALAXY, available at https://usegalaxy.org/ (Afgan et al., 2018). The FastQC (version 0.72.0) (Andrews, 2019) was used for quality control of the raw sequence reads followed by trimming with the Trimmomatic program (version 0.38.0) (Bolger et al., 2014). The sequence reads were *de novo* assembled using Unicycler (version 0.4.8.0) (Wick et al., 2016), and the quality was assessed with Quast (Galaxy Version 5.0.2) (Mikheenko et al., 2018). The draft genome was annotated using the National Center for Biotechnology Information—Prokaryotic Genome Annotation Pipeline (Tatusova et al., 2016) and Rapid Annotations using Subsystems Technology (Aziz et al., 2008). Identification of carbohydrate-active enzymes was carried out by annotating the genome sequence through dbCAN meta server (http://cys.bios.niu.edu/dbCAN2) using HMMER: biosequence analysis with profile hidden Markov models (version: 3.3.1), and all data generated in dbCAN were based on the family classification from the CAZy database (http://www.cazy.org/) (Cantarel et al., 2009; Zhang et al., 2018). The biosynthetic gene clusters for secondary metabolites were determined using Antismash 5.0 (Antibiotics and Secondary Metabolite Analysis Shell) available at https://antismash.secondarymetabolites.org (Kai et al., 2019), BAGEL4 (BActeriocin GEnome mining tooL) available at http://bagel4.molgenrug.nl (Van Heel et al., 2018), and PRISM 4 (Prediction informatics for secondary metabolomes: search a genome for genetically encoded natural products) available at http://magarveylab.ca/prism (Skinnider et al., 2020). Antibiotic resistance genes were identified using the Comprehensive Antibiotic Resistance Database (http://arpcard.mcmaster.ca) (Skinnider et al., 2020). Virulence genes were identified using the virulence factor database (http://www.mgc.ac.cn/VFs/main.htm) (Liu et al., 2019). The genome was assessed for completeness using BUSCO (Benchmarking Universal Single-Copy Orthologs) version 5.0 (Simão et al., 2015), utilizing a lineage dataset of bacilli. Contamination of genome was assessed by CheckM on kbase (https://www.kbase.us/) (Parks et al., 2015).

### Phylogenome Analysis

The genome sequence data were uploaded on the Type (Strain) Genome Server (TYGS), a free bioinformatics platform available at https://tygs.dsmz.de, for a whole genome-based taxonomic analysis (Meier-Kolthoff and Goker, 2019). All pairwise comparisons among the set of genomes were conducted using Genome Blast Distance Phylogeny and accurate intergenomic distances inferred under the algorithm trimming and distance formula *d3.* Average nucleotide identity (ANI) values between the strain and closely related species were calculated with Orthologous Average Nucleotide Identity Tool (OAT) software (Lee et al., 2016).

### *In Vitro* Probiotic Assays

#### Acid and Bile Salt Tolerance

For the determination of bile and acid resistance, a method by Kavitha et al. (2018) was followed with minor modifications. In brief, for acid tolerance, 2% (v/v) of a fresh overnight culture of the isolate was inoculated in NB with varying pH (1–5) values, adjusted with hydrochloric acid, and incubated at 30°C for 24 h. Bacterial cell growth was measured using a spectrophotometer (Biomate 3, Thermo Scientific) at different time intervals (0, 2, 6, and 24 h) at OD600 nm, and the survival percentage of the strain at different pH values was determined. Bile salt tolerance was determined by inoculating 2% (v/v) of overnight culture in NB containing different concentrations of bile salt (0.05, 0.15, 0.3, 1, 2.5, and 5%) and incubated at 30°C shaking at 150 rpm. The survival rate of the isolate was measured as described earlier at 0 and 3 h. Survival of the isolate was represented by percentages (%) survival = (A_1_/A_0_) × 100, where A_1_ is final absorbance, and A_0_ is the initial absorbance.

### Phenol Tolerance Test

Test tubes containing NB were adjusted with different concentrations (0.1 to 0.4%) of phenol. After sterilization, 1% (v/v) of an overnight culture of the strain was inoculated in the test tubes and incubated at 30°C for 24 h. Cell concentration was measured at the absorbance of OD620 nm. Cell viability was estimated by survival percentage (Forhad et al., 2015).

### Lysozyme Tolerance

Lysozyme tolerance was performed following the method described by Yadav et al. (2016) with minor modifications. Briefly, overnight culture (4 ml) was centrifuged at 10,000 rpm for 10 min and washed two times with phosphate-buffered saline solution and suspended in electrolyte A (6.2 g/L NaCl, 2.2 g/L KCl, 0.22 g/L CaCl2, and 1.2 g/L NaHCO3, pH 6.2). To mimic the saliva environment, 1 ml of cell suspension was added to 9 ml of electrolyte A containing 0.01% (w/v) lysozyme (Sigma-Aldrich) and incubated for 20 min (min) at 30°C, at 50 rpm. Bacterial suspension in electrolyte A without lysozyme was included as a control. Cell viability was estimated as log colony-forming units (CFU)/milliliter.

### Gastrointestinal Transit Tolerance

An *in vitro* assay that imitated the gastrointestinal environment was performed following the method of Khalil et al. (2018) with minor modifications. Ten milliliters of overnight bacterial cells were harvested by centrifugation at 10,000 rpm for 10 min. The cells were resuspended in the same volume of electrolyte A (6.2 g/L NaCl, 2.2 g/L KCl, 0.22 g/L CaCl2, and 1.2 g/L NaHCO3) and pH adjusted to 6.2. The tolerance of pepsin was determined by adding 1 ml of bacterial suspension to 9 ml of electrolyte A containing 0.3% (w/v) pepsin in tubes 1–2 and adjusted the pH to 2 and 3, respectively. The samples were incubated for 20 min at 30°C at 50 rpm. Then, 0.10 ml of each sample was plated onto NA plates. To determine the tolerance of pancreatin and bile salt, 1 ml of cell suspension was suspended in 9 ml of electrolyte B (5 g/L NaCl, 0.6 g/L KCL, and 0.3 g/L CaCl2) containing 0.45% (w/v) bile salt and 0.1% pancreatin (Sigma-Aldrich) and adjusted to pH 8. The sample was incubated at 30°C for 120 min, at 50 rpm. The viability of cells was determined by plating 0.10 ml of sample onto NA plates.

An *in vitro* method that mimics the digestive system was used to determine the viability of the bacterial strain through sequential incubation in solutions imitating oral cavity, gastric, and intestinal compartments (Uymaz Tezel, 2019). Briefly, 10 ml of bacterial cells were centrifuged at 10,000 rpm for 10 min, and harvested cells were washed with electrolyte A three times. The cells were resuspended in 10 ml of electrolyte A containing 0.01% (w/v) lysozyme solution for 5 min to mimic the saliva environment. Subsequently, the cells were harvested and resuspended in electrolyte A containing 0.3% (w/v) pepsin at pH 3. The sample was incubated for 90 min at 30°C, shaking at 50 rpm to imitate the gastric environment. Finally, the cells were harvested and resuspended in electrolyte B containing 0.1% pancreatin and 0.45% (w/v) bile salt at pH 8.0, which simulated intestinal digestion conditions. The sample was incubated at 30°C, shaking at 50 rpm for 120 min. Cell viability was assessed through plate counting using the samples collected before and after oral, gastric, and intestinal digestions. Survival rate was calculated *via* colony counts (CFU/milliliter).

### Cell Auto-Aggregation Assay

The assay was conducted following the method by Khalil et al. (2018) and Yadav et al. (2016). Cells were grown for 16–18 h at 30°C and harvested by centrifugation. The cells were washed twice, resuspended in phosphate-buffered saline (PBS) pH 7.4, and adjusted the absorbance to 0.5 at OD600 nm. The suspension was incubated for 5 h at 30°C. Aliquots were taken from the sample at 0 and 5 h; the absorbance was measured at OD600 nm. Auto-aggregation percentage was calculated using the equation: [A_0 h_ - A_5 h_/A_0 h_] ^∗^ 100.

### Co-Aggregation Assay

Strain MHSD3 and eight pathogenic bacteria (*Bacillus cereus* ATCC 10876, *Staphylococcus aureus* NCTC 6571, *Staphylococcus saprophyticus* ATCC 15305, *Escherichia coli* ATCC 25922, *Veillonella parvula* ATCC 10790, *Pseudomonas aeruginosa* ATCC 27853, *Enterococcus faecium* ATCC 13048, and *Klebsiella oxytoca* ATCC 13182) were prepared based on the auto-aggregation assay described earlier. The absorbance of the strain and pathogenic strains were adjusted to 0.5 at OD600 nm. Equal volumes of the strain (1 ml) and pathogenic strains (1 ml) were mixed and incubated at 30°C. The absorbance was measured at OD600 nm at 0 and 5 h of incubation (Khalil et al., 2018). The percentage of co-aggregation was calculated using this formula: [A_0 h_ – A_5 h_/A_0 h_] ^∗^ 100.

### Cell Surface Hydrophobicity

Bacterial cells were harvested and washed twice with PBS, resuspended in 5 ml of PBS, and the optical density was determined at 600 nm. A sample of 2-ml cell suspension was added to 2 ml of different solvents (n-hexadecane, ethyl acetate, and chloroform) and vortexed for 2 min. The aqueous and organic phases were allowed to separate for 30 min at room temperature. One milliliter of the aqueous phase was discarded, and optical density was determined at 600 nm (Khalil et al., 2018). The cell surface hydrophobicity (%) was calculated as (%) = [1 – OD _final_/OD _initial_] × 100.

### Antibiotic Susceptibility

The antibiotic susceptibility and resistance of the strain were assessed using the antibiotic disc diffusion method as described by Yadav et al. (2016). Seven antibiotic discs (Davies Diagnostics, South Africa) were used: erythromycin (15 µg/disc), gentamicin (10 µg/disc), metronidazole (5 µg/disc), polymyxin B (300 units), cefuroxime (30 µg/disc), cefalexin (30 µg/disc), and ciprofloxacin (5 µg/disc) (Singh et al., 2012; Yadav et al., 2016; Somashekaraiah et al., 2019). Overnight culture of strain MHSD3 (100 µl) was spread onto tryptic soy agar plates and allowed to dry. Antibiotic discs were placed on the plates and incubated at 30°C for 24–48 h. The diameter of the zone of inhibition was measured and presented as sensitive, intermediate, and resistant. These results were compared with the interpretative category of zone diameters as described in Performance standards for antimicrobial disc susceptibility Tests by the Clinical and Laboratory Standards Institutes (2020).

### Production of Hydrogen Peroxide

Production of hydrogen peroxide was performed by spotting 10 µl of overnight grown bacterial culture onto NA plates containing 0.5-mM 2,2-azino-bis-3-ethylbenzothiazoline-6-sulfonic acid and 2 mg/L horseradish peroxidase. The plates were incubated for 48–72 h at 30°C in anaerobic conditions. The appearance of a blue halo around colonies was considered a positive test for hydrogen peroxide (Khalil et al., 2018).

### Screening for Exopolysaccharide Production

The bacterial isolate was tested for exopolysaccharide (EPS) production following the method by Khalil et al. (2018). The strain was first streaked on NA plates supplemented with sucrose 5% (w/v) and incubated at 37°C for 72 h. Ropy or mucoid colonies were scored as EPS-producing strains. Next, the strain was grown in NB supplemented with sucrose 5% (w/v) and incubated at 30°C for 72 h. Cells were removed by centrifugation at 8,000 rpm for 15 min at 4°C. The cell-free supernatant was mixed with double volumes of 95% ethanol and allowed to precipitate overnight at 4°C. After incubation, EPSs were separated by centrifugation at 4°C for 30 min at 8,000 rpm. The pellet was dissolved in distilled water and dialyzed using dialysis kits against distilled water for 24 h at 4°C. The pellet was then air-dried for 96 h, and the total weight was measured. The phenol-sulfuric acid assay method was used to determine the concentration of EPS using glucose as a standard (DuBois et al., 1956).

### DNase and Haemolysis Activities

The strain was spot inoculated on a DNase agar medium to check the production of the DNase enzyme. Plates were incubated at 37°C for 48 h. The clear and pinkish zone around colonies was considered positive for DNase activity (Yadav et al., 2016). Overnight culture (50 µl) of the strain was spot inoculated on sheep blood agar and incubated at 30°C for 24 h. The hemolytic activity was distinguished by observing a clear zone of hydrolysis around the colonies (*β*-hemolysis), partial hydrolysis with green-hued zones around colonies (*α-*hemolysis), or no zone around colonies (*γ*-hemolysis). *α*-Hemolysis or *β*-hemolysis indicated positive hemolytic activity, and *γ*-hemolysis was taken as a negative result.

### Extraction of Secondary Metabolites

Extraction of secondary metabolites was carried out using the method by Balachandran et al. (2012). Strain MHSD3 was cultured in a 1,000-ml flask containing 500 ml of Luria Bertani broth and incubated at 30°C at 200 rpm for 7 days. After the 7th day, the culture was centrifuged at 10,000 rpm for 15 min, and the pellet biomass was removed. Equal volumes of chloroform and ethyl acetate (1:1 v/v) were used to extract secondary metabolites from the supernatant. The organic solvent layer was transferred to a clean conical flask and concentrated using a rotary evaporator at 60°C. The extract was dried and used for minimum inhibition concentration and gas chromatography–mass spectrometry analysis.

### Minimum Inhibitory Concentration of Bacterial Crude Extract

The antibacterial activity of strain MHSD3 crude extract was performed by minimum inhibition concentration (MIC) using the method described by Andrews (2001) with minor modifications. The following indicator strains were used: *B. cereus* (ATCC 10876), *Mycobacterium smegmatis* (ATCC 21293), *V. parvula* (ATCC 10790), *E. coli* (ATCC 10536), *P. aeruginosa* (NCTC 10662), *Klebsiella pneumonia* (ATCC 10031), *K. oxytoca* (ATCC 13182), *S. aureus* (ATCC 25923), *S. saprophyticus* (ATCC 15305)*, S. epidermidis* (ATCC 14990), and *E. faecium* (ATCC 13048). The indicator strains were inoculated into Mueller Hinton broth (MHB) for 24 h at 37°C. The inoculums were compared with 0.5 McFarland standard by diluting the culture with MHB. A stock solution of 0.015-g bacterial crude extract was dissolved in 1 ml of dimethyl sulfoxide to make an initial concentration of 15 mg/ml. The serial dilution from stock was carried out using MHB ranging from 15 to 0.23 mg/ml. The 96 well plates were filled with 100-µl indicator strains and 100 µl of different concentrations of crude extracts. Streptomycin antibiotic (Sigma-Aldrich) was used as a positive control and dimethyl sulfoxide as a negative control. All tests were performed in triplicates, and the plates were incubated at 37°C for 24 h. After incubation, 10 µl of 0.02% (w/v) of resazurin sodium salt solution was added to each well as an indicator of microbial growth, and plates were incubated for 2 h at 37°C. A blue color indicated growth inhibition, and a color change from blue to pink indicated bacterial growth.

### Gas Chromatography–Mass Spectrometry Analysis

The crude extract was analyzed on the gas chromatography coupled to a time-of-flight high-resolution mass spectrometry system equipped with an Agilent 7890 A gas chromatograph (Agilent Technologies, Inc., Wilmington, DE, United States) operating in high-resolution, fitted with a Gerstel MPS multipurpose autosampler (Gerstel Inc. Germany) and a Rxi®-5 ms column (30 m × 0.25 mm ID × 0.25 μm) (Restek, Bellefonte, United States). One microliter of each sample was injected in a spitless mode using helium as a carrier gas pumped at a constant flow rate of 1 ml min^−1^. The inlet and transfer line temperatures were 250 and 225°C, respectively. The oven temperature was set at 70°C, held for 0.5 min, ramped at 10°C min^−1^–150°C, held for 2 min, then ramped at 10°C min^−1^ –330°C and held for 3 min for the column to bake out. The MS data acquisition rate was a recommended rate of 13 spectra/s, m/z range of 30–1,000, electron ionization at 70 eV, ion source temperature at 250°C, and a system recommended extraction frequency of 1.25 kHz.

### Identification of Compounds from Gas Chromatography–Mass Spectrometry Data

The compounds present in the crude extract were identified based on the comparison of their mass spectra with those of the National Institute Standard and Technology (https://webbook.nist.gov/chemistry) (Linstrom and Mallard, 2018). Interpretation of functional groups was carried out using the National Center for Biotechnology Information Pubchem database (https://pubchem.ncbi.nlm.nih.gov/) (Kim et al., 2019). Prediction of biological activities was attained with the help of the PASS online database (www.way2drug.com/passonline) (Filimonov et al., 2014) and literature data.

### Statical Analysis

The experiments were performed in triplicates, and results were expressed as mean, standard deviation. Standard errors were calculated and shown in charts as error bars. The significant difference was determined by one-way analysis of variance with *post-hoc* T-tests (Bonferroni Correction) in Microsoft Excel 365. The *p* ≤ 0.05 was considered statistically significant.

## Results and Discussion

### Whole-Genome Sequence of *Bacillus Paranthracis* Strain MHSD3

The draft genome sequence of strain MHSD3 was 5,396 335 bp, with a genomic DNA G + C content of 35.31%, N_50_ of 377,198, and genome coverage of 92×. BUSCO analysis revealed 98.68% of genome completeness (298 completed single-copy genes and 4 duplicated genes). CheckM showed 99.43% genome completeness and 0.57% contamination. The Prokaryotic Genome Annotation Pipeline annotation identified 5,547 genes, of which 5,348 are protein-coding genes, 96 RNA, 87 tRNA, and 5 noncoding RNA (ncRNA) genes (Supplementary Table S1). *B. paranthracis* strain MHSD3 had similar genomic DNA G + C content and genome size compared with other reported *Bacillus* strains.

### Functional Annotation by Rapid Annotations Subsystems Technology

**FIGURE 1:**
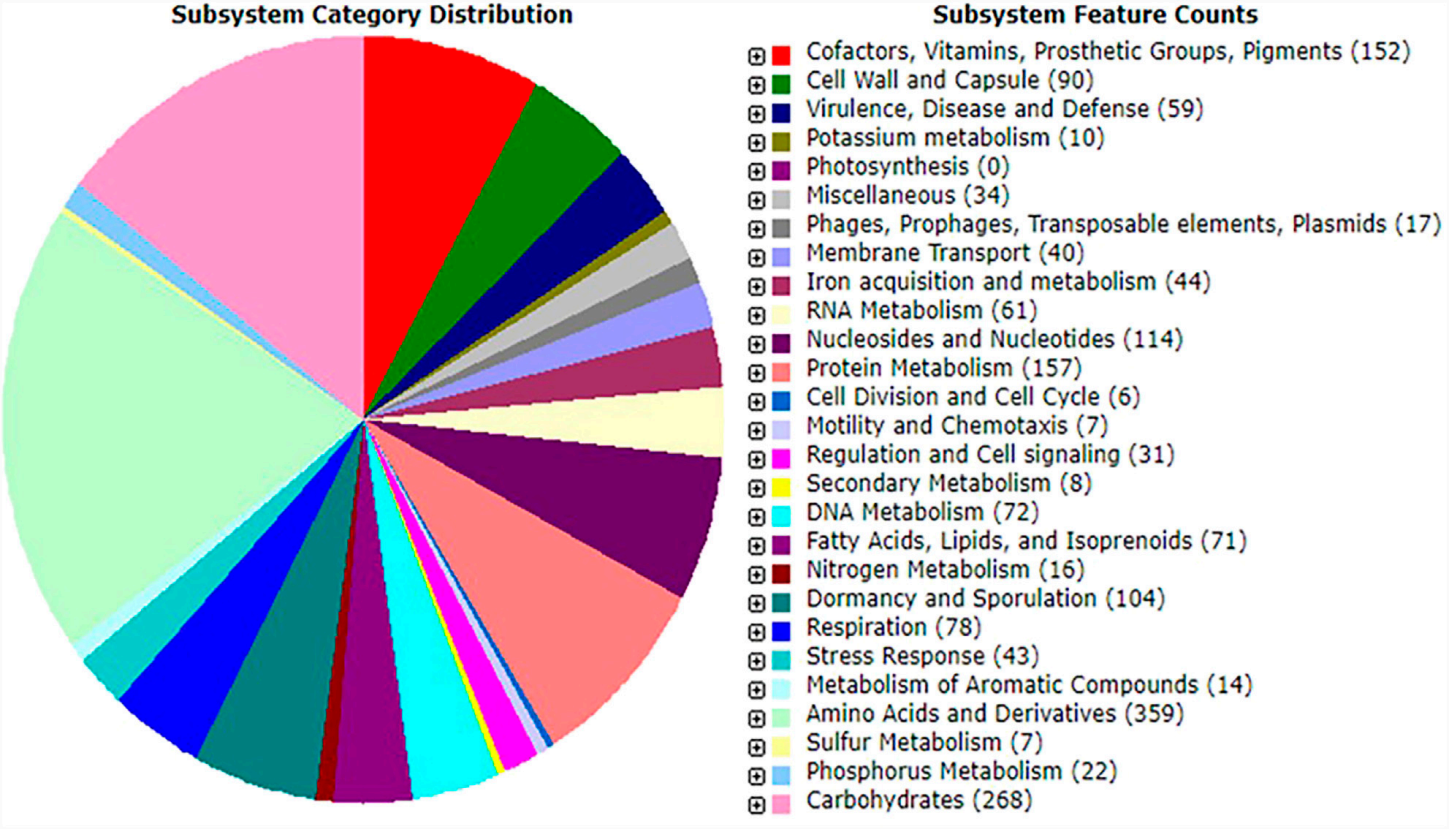
An overview of subsystem categories assigned to genes predicted in genome of *Bacillus paranthracis* strain MHSD3 based on RAST server.

Probiotic species play a vital role in the host gut by synthesizing micronutrients and factors such as amino acids, fatty acids, oligosaccharides, vitamins, and enzymes (Pandey et al., 2015). The Rapid Annotations using Subsystems Technology server-based annotation of strain MHSD3 genome identified a total of 342 functional subsystems (Figure 1), which include genes involved in the synthesis and metabolism of amino acids and derivatives, carbohydrates, fatty acids, lipids, and isoprenoids and co-factors, vitamins, prosthetic groups, pigments with four genes responsible for pyridoxine (vitamin B6) biosynthesis. Pyridoxine (vitamin B6) plays a role in a variety of cellular metabolic processes, including carbohydrates, amino acid, and lipid metabolism (Parra et al., 2018); in addition, it is important in the early development of the nervous system and growth of the embryo (Dalto and Matte, 2017). Pyridoxine has been previously isolated from other probiotic species of the genera *Bifidobacteria*, *Streptococcus*, and *Lactobacillus* (Champagne et al., 2010).

### Carbohydrate-Active Enzyme Analysis

The analysis of carbohydrate-active enzymes revealed that the genome of strain MHSD3 contained 95 genes, 39 glycosyltransferase (GT) genes, 28 glycoside hydrolase (GH) genes, 18 carbohydrates esterase (CE) genes, 5 carbohydrate-binding molecules (CBMs), and 5 auxiliary activities (AA). Further analysis of GH enzyme families in strain MHSD3 revealed the presence of GH13 and GH32, which are reported as major oligosaccharide-degrading enzymes. Oligosaccharides, as complex carbohydrates, are a key source of prebiotics, specifically fructans and galactans have been associated with human gut health (Pokusaeva et al., 2011; Tarrah et al., 2020). Cellulose synthase GT2, a significant enzyme for cellulose biosynthesis, has been identified in the genome MHSD3. It stores cellulose on the cell wall surface as an extracellular matrix for cell adhesion and biofilm formation to protect itself from the surrounding environment (Ghosh et al., 2019). Glycosyltransferases that catalyze the transfer of sugars from the activated donor molecules to specific acceptors are essential for the formation of surface structures recognized by host immune systems (Chung et al., 2018). Thus, the high number of GT genes in strain MHSD3 suggests its probiotic potential, particularly for immune stimulation and pathogen defense.

### Phylogenome Analysis

**FIGURE 2:**
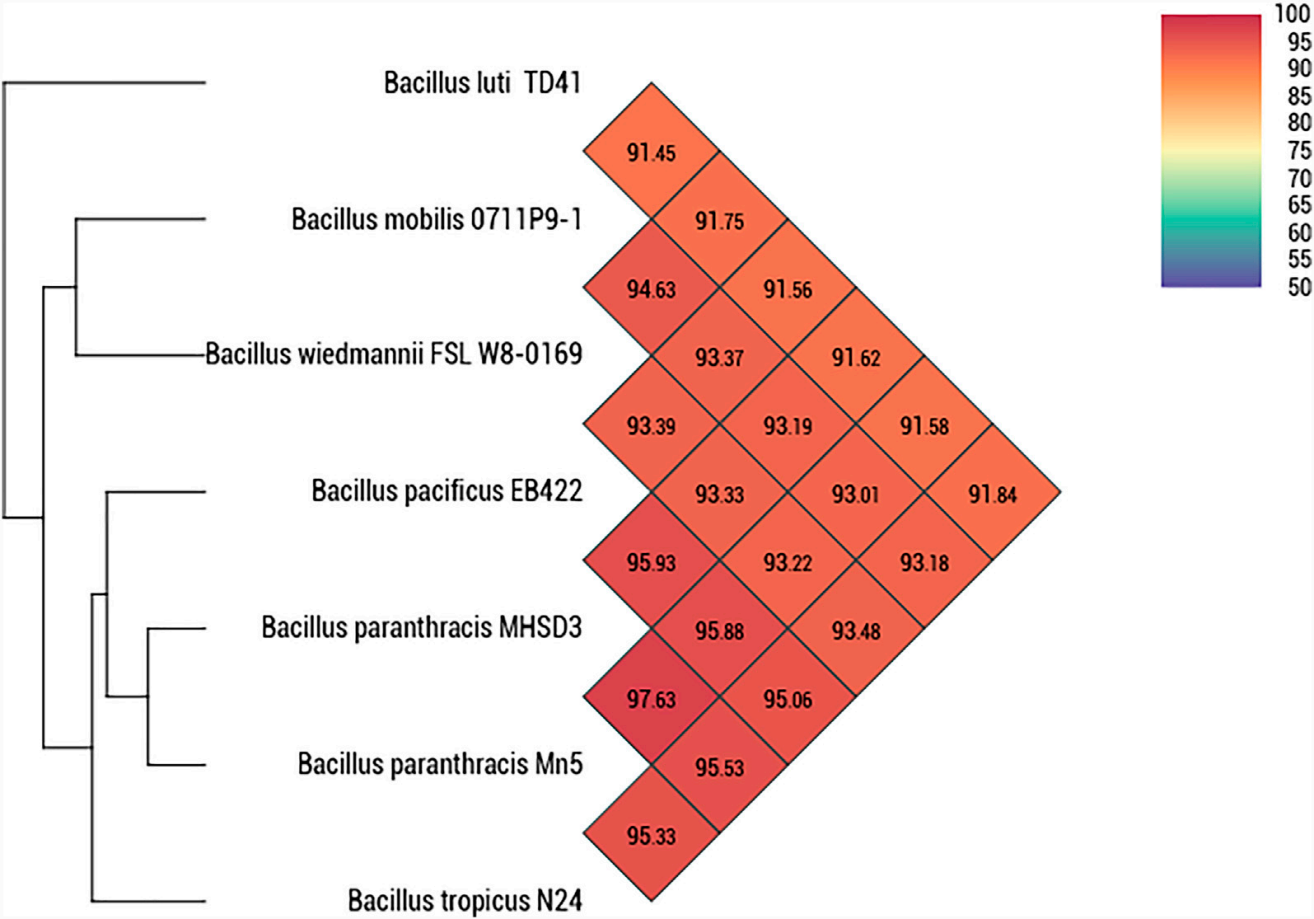
Heatmap generated with OAT software indicating Orthologous Average Nucleotide Identity values calculated between *Bacillus paranthracis* strain MHSD3 and other closely related *B. cereus* species.

A whole genome-based phylogenetic analysis was conducted using Type (Strain) Genome Server (Meier-Kolthoff and Goker, 2019). Strain MHSD3 was closely related to *B. paranthracis* Mn5^T^ with a digital DNA–DNA hybridization (dDDH) value of 79%, which was the highest dDDH value observed with closely related species (Supplementary Table S2). The dDDH value was greater than the recommended cutoff points of 70% for species delineation (Auch et al., 2010). The ANI analysis demonstrated that strain MHSD3 was closest to *B. paranthracis* Mn5^T^ with 97.63% (Figure 2). The observed ANI value was above the species boundary value (ANI > 95–96%) (Lee et al., 2016). Liu et al. (2017) states that an ANI value of 96.2% can be used as a species threshold for the *B. cereus* group. The dDDH and ANI results place MHSD3 within the *B. cereus* group, and the obtained results are congruent. These results indicated that strain MHSD3 should represent a potential subspecies of the species *B. paranthracis* Mn5^T^, as the two had a dDDH similarity value of 79%, which is the highest value for delineating subspecies (Meier-Kolthoff et al., 2014); additionally, strain MHSD3 had an ANI value of 97.63% with *B. paranthracis* Mn5^T^. Further studies are currently underway to fully delineate the strain MHSD3 within the *B. cereus* group and as a subspecies of *B. paranthracis* Mn5^T^.

### Probiotic Genes

The criteria for screening strains as potential probiotic species include assessing functional features such as genes to adhere to host gut, persistence, and survival in harsh conditions (low gastric pH and bile) of the gastrointestinal tract (Ghattargi et al., 2018). The strain must have the ability to antagonize or eliminate pathogens, which is attained by secreting antimicrobial constituents, including bacteriocins and epithelial adhesion sites (Papadimitriou et al., 2015; Oliveira et al., 2017). We describe here genetic features that could impart probiotic properties in *B. paranthracis* strain MHSD3.

### Genes Involved in Stress Response and Bile Salt Resistance

One of the most important characteristics of a bacterium to be considered a probiotic strain depends on its survival mechanism and adaptation or resistance to a low pH environment (Nguyen and Kim, 2018). Studies have shown that probiotic microorganisms consist of genes that aid in toleration or resistance to hostile environments (Nguyen and Kim, 2018). *B. paranthracis* strain MHSD3 genome revealed genes that code for stress response, adhesion, lactate synthesis, metabolic rearrangement, and transcriptional regulators, all of which play a role in survival in low pH and reduction of pathogen colonization in a gut environment (Table 1). The presence of adhesins in the probiotic cell wall plays a significant role in the adhesion of the strain to the intestine (Monteagudo-Mera et al., 2019). Six adhesion genes were identified from the annotated genome. The sortase-dependent surface proteins play a role in processes associated with mucosal adhesion and function in some parts of the maintenance of intestinal homeostasis (Muñoz-Provencio et al., 2012). Sortase class A (*srtA*) is responsible for the covalent anchoring of the LPXTG proteins to the cell wall. Some LPXTG proteins, particularly those carrying mucus binding domains, play a role in adhesion to host surfaces (Marraffini et al., 2006). Sortase C (*srtC*) plays a crucial role in pilus assembly and is responsible for anchoring the pilus to cell wall peptidoglycan of Gram-positive microorganisms. The pili formed by class C sortases are responsible for adherence to epithelial cells and extracellular matrix proteins, and interaction with the host immune system (Call and Klaehammer, 2013). Mucus-binding proteins, surface proteins such as fibronectin-binding proteins, and surface-layer proteins contribute to the adherence of bacteria to the intestinal mucosa (Lehri et al., 2015; Hymes et al., 2016). *B. paranthracis* MHSD3 harbors genes coding for fibronectin-binding protein such as *fbpA* and mucus-binding protein *lpsA.*

**TABLE 1:** Potential genes related to different probiotic properties from *Bacillus paranthracis* strain MHSD3 genome.

Function	Genes	Gene product
Modulation of the immune system/Acid stress	*clpB*	Potential immunogenic proteins
Production of nutrients and other beneficial processes	*ccpA*	Catabolite control protein A
Influencing blood cholesterol		
Adhesion	*srtA*	Class A sortase
	*srtC*	class C sortase
	*fbpA*	Fur-regulated basic FbpA
	*lpsA*	Lipoprotein signal peptidase LspA
	*dltD*	Methionine sulfoxide reductase-Alanylation of LTA
	*dltA*	d-Alanylation of LTA
Acid stress	*atpC*	F0F1 ATP synthase subunit epsilon
	*atpD*	F0F1 ATP synthase subunit beta
	*atpG*	F0F1 ATP synthase subunit gamma
	*atpH*	F0F1 ATP synthase subunit delta
	*atpF*	F0F1 ATP synthase subunit B
	*atpB*	F0F1 ATP synthase subunit C
	*atpE*	F0F1 ATP synthase subunit C
	*recA*	Recombinase RecA
	*soda*	Speroxide dismutase [Mn]
	*relA*	GTP diphosphokinase
	*groES*	Co-chaperone GroES
	*groEL*	Chaperonin GroEL
	*recA*	Recombinase RecA aspartate-tRNA ligase
	*aspS*	
Acid stress/bile resistance	*gpmA*	2,3-diphosphoglycerate-dependent phosphoglycerate mutase
	*dnaK*	Chaperone protein DnaK
	*dnaJ*	Chaperone protein DnaJ
	*glmU*	Bifunctional UDP-N-acetylglucosamine diphosphorylase/glucosamine phosphate
	*bshA*	N-acetyl-alpha-D-glucosaminyl l-malate synthase BshA
	*bshB*	Bacillithiol biosynthesis deacetylase BshB1
	*bshC*	Bacillithiol biosynthesis cysteine-adding enzyme BshC
	*luxS*	S-ribosylhomocysteine lyase LuxS
Bile resistance	*nagB*	Glucosamine-6-phosphate deaminase
	*pyrG*	CTP synthase
	*argS*	Arginine-tRNA ligase
	*rpsC*	30S ribosomal protein S3
	*rpsE*	30S ribosomal protein S5
	*rplD*	50S ribosomal protein L4
	*rplE*	50S ribosomal protein L5
	*rplF*	50S ribosomal protein L6
Antibiotic resistance	*rpoB*	DNA-directed RNA polymerase subunit beta
	*mecA*	Adaptor protein MecA
	*uppP*	Bacitracin resistance undecaprenyl-diphosphatase
	*bacA*	Bacitracin resistance
	*bla*	Class A beta-lactamase Bla1
	*groEL*	Chaperonin GroEL
	*fosB*	FosB/FosD family fosfomycin resistance bacillithiol transferase
Fatty acid synthesis	*fabD*	ACP S-malonyltransferase
	*fabH*	Beta-ketoacyl-ACP synthase III
	*fabF*	Beta-ketoacyl-ACP synthase II
	*fabI*	Enoyl-ACP reductase FabI
	*accC*	Acetyl-CoA carboxylase biotin carboxylase subunit
Lactate synthesis	*mdh*	Malate dehydrogenase
Others	*dapA*	4-hydroxy-tetrahydrodipicolinate synthase
	*dps*	DNA protection during starvation and other stresses
Transcriptional regulator	*sigB*	RNA polymerase sigma factor SigB
	*ctsR*	Transcriptional regulator CtsR
	*hrcA*	Heat-inducible transcriptional repressor HrcA
Metabolic rearrangement	*alsD*	Alpha-acetolactate decarboxylase

Five genes coding for toxin–antitoxin systems were also identified; these are believed to play a role in stress response (Wei et al., 2016). A total of 30 genes responsible for acid and bile salt stress were identified (Table 2). The F_1_F_0_-ATPase is encoded by the *atp* operon, which in most microbes, it consists of these genes: *atpB*, *atpE*, *atpF*, *atpH*, *atpA*, *atpG*, *atpD*, and *atpC* (Ventura et al., 2004). All genes were identified in the genome except for *atpA.* The *atp* genes are essential for the host microorganisms to survive or tolerate an acidic environment. The “atp” operon is mainly associated with the pumping of protons from the bacterial cytoplasm to the outside and therefore helps in maintaining neutral pH in the bacterial cytosol (Duary et al., 2010). S-Ribosylhomocysteinase (*LuxS*) is a key enzyme for the biosynthesis of Autoinducer-2. The Autoinducer-2 has been reported to enhance bacterial stress resistance (Laganenka et al., 2016), and it is involved in responding to environmental stress and regulating growth and metabolism (Liu et al., 2018). The *luxS* gene is linked with acid tolerance and the ability to adhere to intestinal epidermal cells in some of the probiotic strains, *Lactobacillus rhamnosus* and *Lactobacillus acidophilus* (Jia et al., 2018). These findings indicate that the *B. paranthracis* strain MHSD3 possesses potential probiotic properties.

**TABLE 2:** Identified secondary metabolite gene clusters in *Bacillus paranthracis* strain MHSD3 using anti-SMASH, BAGEL 4, and PRISM.

Cluster	Type	Most similar known cluster	Percentage %	Other species where clusters were identified	Functions		References
1	NRPS	Bacillibactin	46	*Bacillus subtilis*	Antibacterial, Antifungal	Fazel Rabbee and Baek (2020)	
2	Beta-lactone	Fengycin	40	*Bacillus velezensis* FZB42	Antifungal, Antibacterial	Medeot et al. (2020)	
3	Terpene	Molybdenum factor	17	*Staphylococcus carnosus*	-	-	
4	NRPS	Thailanstatin A	10	*Burkholderia thailandensis*	Anticancer, Antiproliferative	Liu et al. (2016)	
5	Bacteriocin	-	-	-	Antimicrobial	Lv et al. (2020)	
6	LAPs	-	23.71	*Methanocaldococcus jannaschii* ATCC 43067	Antimicrobial	Arnison et al. (2013)	
7	Sactipeptides	-	76.55	*Bacillus subtilis* 168	Antibacterial, hemolytic properties	Chen et al. (2021)	
8	Lanthipeptide classIII/IV	Lactocin 705	-	*Lactobacillus casei CRL 705*	Antimicrobial	Vignolo et al. (1993), Cuozzo et al. (2000)	
9	Bacterial head-to-tail cyclized peptide	**-**	**-**	*Bacillus pumilus B4107*	Antimicrobial	van Heel et al. (2017)	

LAPs = linear azole-containing peptides; NRPS = non-ribosomal peptide synthetase.

### Antibiotic Resistance Genes

Antibiotic resistance is common in bacterial species, and it is conferred by resistance genes acquired through horizontal gene transfer of plasmids, uptake of foreign DNA material from the environment, and mutations that might occur on bacterial chromosomal DNA (Martinez and Baquero, 2000). Although it is ideal for probiotic strains to carry a limited number of antibiotic resistance genes so as not to be the source for transferring these genes to other gut bacteria, especially pathogens (Gueimonde et al., 2013), resistance to some antibiotics is required, as probiotics get administered with antibiotics (Khatri et al., 2019). Bacitracin resistance genes (*bacA* and *uppP*) and fosfomycin resistance bacillithiol transferase gene (*fosB*) were identified in the genome of MHSD3. Bacitracin is a cyclic polypeptide antibiotic produced by certain *Bacillus* species (Ma et al., 2019). Bacillithiol is a compound found in *Bacillus* sp., which is involved in microbial resistance to the antibiotic fosfomycin (Roberts et al., 2013). Class A beta-lactamase was also identified, which indicates there could be resistance against penicillin and cephalosporins in *B. paranthracis* strain MHSD3. We did not identify any beta-lactamase resistance genes reported in other *Bacillus* probiotic species such as *Bacillus clausii* strains (Girlich et al., 2007; Khatri et al., 2019).

### Biosynthetic Gene Clusters and Bacteriocins

The antiSMASH database revealed six antimicrobial gene clusters, including non-ribosomal peptide synthetases (NRPS), beta-lactone, bacteriocins, and linear azole-containing peptides. The PRISM database discovered three antimicrobial gene clusters, which include class III/IV lantipeptide, bacterial head-to-tail cyclized peptide, non-ribosomal peptide; and the BAGEL4 discovered three gene clusters, which included linear azole-containing peptides, sactipeptides, and lantipeptide_class_IV (Table 2). The biosynthetic gene clusters mentioned earlier have been reported to have antimicrobial activities against bacterial and fungal pathogens (Wu et al., 2018). Lanthipeptides are reported to exhibit a wide range of bioactivities spanning from antimicrobial activities to antiviral, antinociceptive, and antiallodynic (Balty et al., 2019). One of the lanthipeptides_class_IV open reading frames showed homology to a known bacteriocin, lactocin 705, a bacteriocin produced by *Lactobacillus casei* CRL 705, which is a probiotic strain (Vignolo et al., 1993; Cuozzo et al., 2000).

Bacteriocins are ribosomally synthesized antimicrobial peptide molecules secreted by bacteria that have antibacterial activity against competitive, similar or related bacterial strains (Heng and Tagg, 2006; Chopra et al., 2014; Gadde et al., 2017). Bacteriocins have been used as food preservatives (Gadde et al., 2017), as they were mostly produced by food-grade lactic acid bacteria and were used for the prevention of specific bacterial strains in food (Cotter et al., 2005). Thus, the production of bacteriocins is considered an important feature when selecting probiotic strains (Gadde et al., 2017). We identified two genes coding for sonorensin and thiazole-containing bacteriocins. Sonorensin bacteriocin belongs to the heterocycloanthracin subfamily and was recently discovered from *B. sonorensis* MT93^T^ (Chopra et al., 2014). Sonorensin has been reported to be effective as a bio-preservative of fruit products (Chopra et al., 2015), meat (Chopra et al., 2015), and shelf-life extender of pasteurized milk (Chopra et al., 2015). Additionally, it is known to prevent the biofilm formation of *S. aureus* (Chopra et al., 2015). Thiazole is a heterocycle of thiazole/oxazole-modified microcins that are a class I bacteriocin with antibacterial activity (Baquero et al., 2019), and are ribosomally synthesized and posttranslationally modified peptides (RiPPs) (Baquero et al., 2019). Thiazole exists in gene clusters that code for various factors involved in transport, modification, and immunity (Collins et al., 2017). The presence of these two bacteriocins in the strain MHSD3 genome indicates its important role as a probiotic and a role in fighting pathogens.

### Virulence- and Pathogenic-Associated Genes

For strains earmarked for probiotic use, genome sequences are essential for thorough safety evaluations (Wassenaar et al., 2015). In addition to screening the genome for probiotic potential, strains should also be screened for virulence-, pathogenic-, and toxin-associated genes. Such genes should not be in probiotic species (Wassenaar et al., 2015). We identified two nonhemolytic enterotoxin genes, *nheA* and *nheB*, coding for nonhemolytic enterotoxin NHE subunit A and nonhemolytic enterotoxin NHE subunit B, respectively. These two nonhemolysins were previously identified in *Bacillus* species deemed pathogens (Arnesen et al., 2008; Rossi et al., 2018) and probiotics (Jiménez et al., 2013; Johnson et al., 2015; Li et al., 2018). The identification of enterotoxin genes in both pathogen and probiotics indicate that some of the virulent genes are often conserved in pathogens, commensals, and probiotics (Wassenaar et al., 2015). This study is consistent with previous reports where virulent genes have been identified in probiotics such as *Bacillus toyonensis* (Jiménez et al., 2013), *Bacillus coagulans* (Johnson et al., 2015), and *E. coli* (Willenbrock et al., 2007). The genome does not possess genes coding for cereulide synthetase, enterotoxin FM, and cytotoxin K, which are commonly present in food poisoning pathogens such *B. cereus* and *Bacillus thuringiensis* (Li et al., 2018). *B. paranthracis* ICIS-279 with probiotic prospects was previously reported from human intestines (Bukharin et al., 2019). These genomic findings indicate the probiotic potential of strain MHSD3 subject to further *in vivo* investigations on its suitability for use as a probiotic.

A total of 18 genes related to virulence factors were detected from the virulence factor database. Most of these genes are associated with cell function and defense, such as exopolysaccharides (*bpsC*), capsular polysaccharides (*rmlB*), and bacillibactin genes (*dhbA*, *dhbB*, *dhbC*, *dhbE*, *dhbF*, *hal*, and *ilsA)*. Exopolysaccharides play a role in cell adhesion during abiotic or biotic surfaces (Caro-Astorga et al., 2020). It can help bacteria survive osmotic, desiccation, and oxidative stress conditions (Liu et al., 2017), and play a role in cryoprotection and biofilm formation (Casillo et al., 2017). Polysaccharides are involved in discovering the strain-specific properties important for probiotic action, such as stress resistance, adhesion, and the defense mechanism of the host (Lebeer et al., 2009). Capsular polysaccharides have been discovered in the colonization of the digestive tract by bacteria from the genus *Bacteroides*, and they play a role in modulating the immune system (Porter et al., 2017). Bacillibactin produced by *Bifidobacterium* species was confirmed to adapt under the iron limiting environment of the gastrointestinal tract (Soni et al., 2020). Most microbial virulence factors are associated with pathogens. However, virulence factors, such as adhesions, were also encoded in the genomes of commensal bacterial (Sui et al., 2009).

### Genes Involved in Endophytic Lifestyle

We identified several putative genes involved in bacterial endophyte lifestyle (Supplementary Table S3). These genes are putatively involved in secretion and delivery systems, transport, transcriptional regulators, and plant polymer degradation or modification. Transcriptional regulator, the LysR family, regulates an adverse set of genes mainly involved in bacterial virulence, motility, metabolism, and quorum sensing. The AraC family regulators are responsible for carbon metabolites, stress response, and virulence management (Ali et al., 2014). Transcriptional regulator, the LysR family, was also identified in *Burkholderia phytofirmans* PsJN, which is a bacterial endophyte associated with plant growth-promoting activity (Weilharter et al., 2011; Ali et al., 2014).

Four genes are responsible for transporting different molecules across membranes**.** The transport genes allow endophytes to take up plant-synthesized nutrients that may be available in the plant (Taghavi et al., 2010). Another class of genes code for secretion and delivery system. This gene plays an important role in infection, virulence, and pathogenicity. Type I and II secretion systems are present in numerous endophytes, and type III and IV secretion systems are mostly found in bacterial pathogens (Reinhold-Hurek and Hurek, 2011).

Cupin and hydrolase enzyme have been reported to be present in various endophytic bacteria (Dias et al., 2019). Hydrolase enzymes play a role in the diversity of sugar utilization, which might be considered a useful factor for a competent endophyte (Dunwell and Khuri, 2004; Ali et al., 2014). Cupin superfamily of proteins plays a role in the modification of plant cell wall carbohydrates. The presence of reductase-related genes was also identified. These genes function in protecting endophytes during oxidative stress (Ali et al., 2014).

### *In Vitro* Probiotic Assays

#### Acid, Bile Salt, and Phenol Tolerance

**FIGURE 3:**
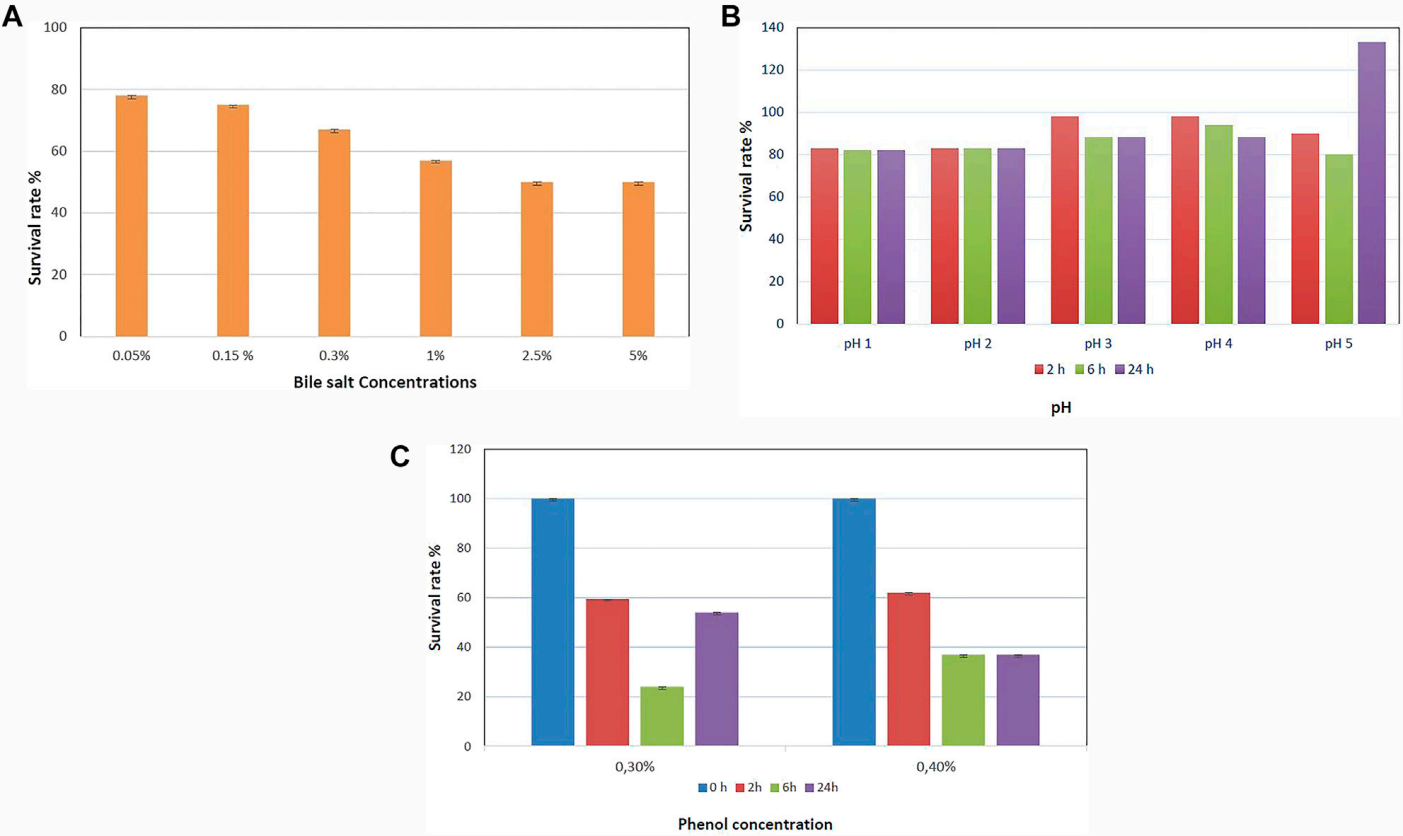
**(A)** Survival rate of *Bacillus paranthracis* strain MHSD3 at different concentrations of bile salt, **(B)** different pH, and **(C)** different phenol concentrations.

The survival rates of *B. paranthracis* MHSD3 in bile salt and acid conditions are shown in Figures 3A,B, respectively. *B. paranthracis* MHSD3 was able to survive in all bile salt concentrations with a survival rate of 50–78% at 5 and 0.05% bile salts, respectively. Li et al. (2018) recorded a survival rate of 59.67% at 0.3% for *Bacillus* sp. DU-106. Strain MHD3 was further able to tolerate all pH levels with a survival rate ranging from 82 to 133% at pH 1 and pH 5, respectively. A similar observation was made in a study by Li et al. (2018), where the survival rate for *Bacillus* sp. at pH 4.94 was 96.77%, whereas at pH 1.55 was 7.14%. *B. paranthracis* strain MHSD3 showed a higher survival rate (133%) at pH 5 because, as indicated in the current study and study by Li et al. (2018), increased pH value results in increased growth or survival of the strains, resulting in increased OD readings. Probiotic species must survive in low pH in the stomach and the bile salt in the small intestine. These results showed that strain MSHD3 has potential probiotic properties. Resistance to phenol is another important criterion for probiotic strains, as the gut bacteria can deaminate some of the diet-derived amino acids leading to the formation of phenols (Sathyabama et al., 2015). *B. paranthracis* MHSD3 showed different sensitivity toward phenol concentrations. The strain was able to tolerate 0.1 and 0.2% phenol concentrations at OD600, and with increasing phenol concentration, strain MHSD3 was more sensitive toward 0.4% phenol concentration and had a survival rate of 37% (Figure 3C).

### Gastrointestinal Transit Tolerance

When probiotics are orally administered, they are first exposed to saliva, which contains an antibacterial enzyme called lysozyme. Secondly, they transit through the stomach to the small intestine and colon (Han et al., 2021). In the present study, *B. paranthracis* strain MHSD3 was evaluated in both gastric and intestinal juices. Strain MHSD3 showed no reduction of cells when exposed to lysozyme for 5 and 20 min as compared with control (Table 3). When the cells were stimulated with gastric juices at pHs 2 and 3, the viability dropped by 0.6 log CFU. When stimulated with intestinal juices, the *B. paranthracis* strain MHSD3 showed a log reduction of 2.4. The strain, when exposed to conditions simulating the digestive tract system, showed a reduction in viability in the gastric environment by log CFU of 2.1 compared with the log CFU in the saliva environment. When the cells were treated with 0.01% pancreatin and 0.45% bile salt at pH 8 to stimulate the intestinal environment, the strain showed loss of cell viability by 4.3 log CFU compared with the saliva environment.

**TABLE 3:** Gastrointestinal transit tolerance of *Bacillus paranthracis* MHSD3.

GIT	Time (min)	Log CFU/ml
**Control**	20	8.0 ± 0.25^a^
**Stimulated oral cavity**		
0.01% Lysozyme	20	8.0 ± 0.48^a^
**Stimulated gastric juice**		
0.3% (w/v) pepsin, pH 2	20	7.2 ± 0.05^a^
0.3% (w/v) pepsin, pH 3	20	7.2 ± 0.09^a^
**Stimulated intestinal juices**		
0.01% Pancreatin (w/v), Bile salt 0.45%	120	5.4 ± 0.02^a^
**Stimulated digestive system**		
**Saliva environment**	5	8.4 ± 0.03^a^
**Gastric environment**	90	6.3 ± 0.04^a^
**Intestinal environment**	120	4.1 ± 0.12^a^

a Indicates standard deviation of three replicates. The values with the same superscript are not significantly different (*p* ≥ 0.05).

### Cell Auto-Aggregation, Co-Aggregation, and Hydrophobicity

Cell adhesion is a significant prerequisite for probiotic bacteria, as it prevents pathogen invasion and inflammation in the intestinal tract, and it protects intestinal epithelial cells (Krausova et al., 2019; Guan et al., 2020). The probiotic strains must adhere to the mucosal surface in the gastrointestinal tract. According to Wang et al. (2010) and Khalil et al. (2018), a good auto-aggregation ability should be greater than 40%, and any strain with less than 10% is considered to have weak auto-aggregation. *B. paranthracis* strain MHSD3 was regarded to have a good auto-aggregation ability with a value of 79% (Supplementary Table S4). In addition, chloroform, ethyl acetate, and hexadecane were used to test the hydrocarbon affinity of *B. paranthracis* MHSD3. Strain MHSD3 showed much higher hydrophobicity activity (54.28%); chloroform and ethyl acetate were the lowest with 20.18% (Supplementary Table S4). The strain was able to co-aggregate all pathogenic strains tested. The highest co-aggregation was 44.74% with *S. aureu*s, and the lowest was at 15.04% with *V. parvula* (Table 4). Co-aggregation traits are important criteria for food preservation and have an impact on eliminating pathogens (Xu et al., 2009). Surface hydrophobicity, auto-aggregation, and co-aggregation are properties that provide a great advantage for microbial colonization in the intestinal tract (Pan et al., 2017).

**TABLE 4:** Co-aggregation assay *of Bacillus paranthracis* MHSD3.

Pathogenic strains	Survival rate (%)
*Escherichia coli*	41.46 ± 0.47
*Staphylococcus aureus*	44.74 ± 0.62
*Pseudomonas aeruginosa*	39.04 ± 0.07
*Veillonella parvula*	15.04 ± 2.43
*Klebsiella oxytoca*	44.30 ± 0.20
*Staphylococcus saprophyticus*	32.23 ± 1.04
*Bacillus cereus*	38.46 ± 0.62
*Enterococcus faecium*	30.22 ± 0.82

Values are means of triplicate measurements with ±standard deviation.

### Antibiotics Susceptibility

*B. paranthracis* MHSD3 showed resistance to polymyxin B and metronidazole. Polymyxin B has a narrow spectrum activity, and it has no activity against Gram-positive bacteria and anaerobic bacteria (Zavascki et al., 2007). Metronidazole also has a limited spectrum of activity. It is highly active against Gram-positive and Gram-negative anaerobic bacteria (Löfmark et al., 2010). MHSD3 exhibited sensitivity to cefalexin, ciprofloxacin, and erythromycin and was moderately susceptible to gentamicin and cefuroxime (Supplementary Table S5). Generally, candidate probiotic strains should be safe (Gueimonde et al., 2013) and exhibit little or no antibiotic resistance. Therefore, *B. paranthracis* strain MHSD3 is a potential probiotic strain, as it exhibited sensitivity to most antibiotics used in this study.

### Production of Exopolysaccharides and Hydrogen Peroxide

*B. paranthracis* MHSD3 produced cream white ropy colonies due to EPS production. Further, the phenol–sulfuric method was used to calculate the concentration of the EPS using the glucose standard curve. The EPS production of *B. paranthracis* MHSD3 was 65 mg/L when using sucrose as a carbon source. Bacterial species that produce EPS have better chances of surviving the hostile environment in the gut (Patel et al., 2012). Furthermore, EPS production by probiotic bacteria has various functions, such as biofilm formation, allowing colonization of bacteria on the intestinal epithelial cell surfaces through cell–cell interactions, quorum sensing, and prevention of pathogenic bacterial growth (Vinothkanna et al., 2021). Exopolysaccharides produced by bacteria can be beneficial in the pharmaceutical sector due to their immunostimulatory, immunomodulatory, antitumor, antiviral, anti-inflammatory, and antioxidant properties (Abdhul et al., 2014). Hydrogen peroxide is used in food products to prolong their shelf-life because of its inhibitory effects on other microorganisms (Yadav et al., 2016). Strain MHSD3 did not show the ability to produce hydrogen peroxide.

### Minimum Inhibition Concentration

The MIC of *B. paranthracis* strain MHSD3 ranged between 1.87 and 15 mg/ml (Supplementary Table S6). *Staphylococcus epidermis and S. saprophyticus* had the lowest MIC of 1.87 mg/ml, followed by *K. pneumonia and V. parvula* with MIC of 3.75 mg/ml. These MIC values are much higher than the expected value of plant extract, which is <0.1 mg/ml (Eloff, 2004; Ríos and Recio, 2005). A few studies have reported probiotics with MIC values higher than the expected MIC value of plant extracts. Nyanzi et al. (2014) reported an MIC value of 1.2–2.5 mg/ml of various antifungal methanol extracts from probiotic bacterial cells tested against *Candida albicans*. The MIC value from various *Lactobacillus* species against *E. coli* ATCC 8739, *E. coli* ATCC 11775, *S. aureus* ATCC 6538, *S. aureus* ATCC 12600, and *Salmonella typhimurium* ATCC 14028 were reported to be 1.25–5 mg/ml (Nyanzi et al., 2015). Ström (2005) reported that cyclo (L-Phe-Pro) extracted from *Lactobacillus plantarum* MiLA had an MIC value of 20 mg/ml when tested against *Aspergillus fumigatus* and *Penicillium roqueforti.*

### Hemolysis and DNase Activities

For a strain to be considered probiotic, it should be assessed for the presence of virulence factors to determine what potential risks might be involved in its use. *B. paranthracis* strain MHSD3 showed no DNase-producing ability when grown on DNase agar media for 24 h. However, the strain showed *β*-hemolysis when grown on 5% sheep blood agar plates. Based on previous study, three commercial *Bacillus* probiotics (Bactisubtil, Subtyl, and Biosubtyl) produced *β*-hemolysis on 5% sheep blood (Hoa et al., 2000). Additionally*,* commercial probiotics *B. toyonensis* BCT-7112^T^, a member of the *B. cereus* group, and *B. coagulans* ATCC 7050^T^ have been reported to contain non-hemolytic enterotoxin hemolysin (Li et al., 2018; Abdulmawjood et al., 2019). Duc et al. (2004) reported that enterotoxins are not always produced, and the microenvironment (luminal pH, adhesion, and competition with other commensal bacteria, food consumption) within the gut may affect the production of enterotoxin, which may result in food poisoning not occurring*.*

### Gas Chromatography–Mass Spectrometry Analysis of Bioactive Metabolites Produced by *Bacillus Paranthracis* MHSD3

The chromatogram (Supplementary Figure S1) predicted the presence of numerous compounds, which were identified according to their retention time, peak area, and molecular formula. The retention time, the abundance of the compounds, and the biological properties are shown in Table 5. The nature of compounds identified included esters, amines, alkanes, amino acids, alkaloids, amides, ketones, vitamins, and phenolic compounds, which are reportedly secreted by probiotics (Jin et al., 2019) and bacterial endophytes (Singh et al., 2017). In total, more than 30 volatile compounds were identified from strain MHSD3 extracts (Table 5), although not a high number, this could be due to environmental growth conditions such as pH and growth media that affect the biosynthesis of secondary metabolites (Giubergia et al., 2016; Kjærbølling et al., 2019), and cryptic gene clusters not expressed under current laboratory conditions (Doroghazi and Metcalf, 2013); additionally, method and equipment used for extraction and identification, respectively, can affect the number of metabolites identified (Gbashi et al., 2017). Organic acids such as benzoic acid, 4-butoxy-3-methoxy-, perhydro-1-quinolizinylmethyl ester, and benzoic acid, 4-ethoxy-, ethyl ester were identified from the *B. paranthracis* strain MHSD3 extract. Benzoic acid, 4-ethoxy-, ethyl ester have been reported to confer antimicrobial activity (Mujeeb et al., 2014; Wei et al., 2018). Probiotic bacteria are known to produce secondary metabolites such as oligosaccharides, organic acids, antimicrobial peptides, and digestive enzymes during fermentation. All these metabolites play a vital role in the rebalance of the microbiota and osmotic pressure of the intestine, enhancement of nutrient digestion and improvement, anti-stress, and prevention of obesity (He et al., 2016).

**TABLE 5:** Bioactive compounds identified from *Bacillus paranthracis* strain MHSD3 secondary metabolites crude extract by GC-MS analysis.

Nature of compound	RT (min/sec)	Compounds	Area value	Biological activity
**Ester**	8.00	Butanoic acid, 2,3-dihydroxypropyl ester	1,263,946	Anti-inflammatory, cholesterol antagonist, anti-hypoxic, anti-eczematic, anti-ulcerative, and anticancer activities
	8.90	Methyl anthranilate	296,399	Antinociceptive, anti-seborrheic, and analgesic activities
	9.31	Propanoic acid, 2-methyl-, 2-ethyl-3-hydroxyhexyl ester	344,640	Antibacterial cholesterol antagonist, antiviral, antifungal, and antioxidant activities
	9.75	Mandelic acid, 3,4-dimethoxy-, methyl ester	19,835	Antibacterial, anti-seborrheic, and anti-aging activities
	11.94	Benzoic acid, 4-ethoxy-, ethyl ester	469,122	Antimicrobial, antioxidant, anti-seborrheic, anti-eczematic, cholesterol antagonist, and antipyretic activities
	13.06	Diethyl Phthalate	1,253,729	Industrial uses (plastic), insecticides, anti-seborrheic and anti-eczematic
	14.05	4-Oxo-4-phenylbutyric acid, heptyl ester	41,878	NF
	15.51	Benzyl Benzoate	322,262	Acaricide, scabicide, and pediculicide activities
	15.63	Tridecanoic Acid, methyl ester	88,495	Anthelminthic, anti-inflammatory, antimicrobial, and anticancerous activities
	17.23	Undecanoic acid, methyl ester	184,648	Antifungal activity
	18.28	Benzoic acid, 4-butoxy-3-methoxy-, perhydro-1-quinolizinylmethyl ester	362,755	Anti-amyloidogenic and anti-eczematic
	22.33	Phosphoric acid, tris(2-ethylhexyl) ester	32,827,025	Antimicrobial, vasoprotector, and analeptic activities
	22.94	Phthalic acid, heptadecyl 2-propylpentyl ester	10,842,537	Spasmolytic, fibrinolytic, and pesticide activities
	24.40	1,3-Benzenedicarboxylic acid, bis(2-ethylhexyl) ester	1,134,776	Antimicrobial activity
	24.80	Sebacic acid, di (4-octyl) ester	75,310	Antibacterial, anti-ischemic, antiviral, anti-hypoxic, and anti-seborrheic activities
**Alkanes**	5.42	Tridecane	3,164,308	Antimicrobial, anti-eczematic, and anti-neurotic activities
	9.21	Dodecane, 2,7,10-trimethyl-	817,683	Antimicrobial and antioxidant activities
	9.57	Hexadecane	2,680,770	Antibacterial, antifungal, and antioxidant activities
	10.95	Cyclopropane	179,698	Insecticidal, antifungal, herbicidal, antibacterial, antitumor, and antiviral activities
	23.22	Heptacosane	554,978	Antibacterial activity
**Amino acids**	3.75	l-Proline	1,211,411	Nutrition, wound healing, antioxidative reactions, and immune responses
	10.52	Tyrosine	10,818,597	NF
	12.98	3-Phenylaniline	65,017	Nutritional supplement
	16.21	dl-Alanyl-l-leucine	8,878,565	NF
	16.71	l-Proline, N-valeryl-, decyl ester	1,211,411	NF
	18.32	3-Aminocarbazole	83,203	Pesticides
	19.17	d-Norleucine	558,168	NF
	25.07	Phenylalanine, 2,5-diketo-3-hydroxy-6-piperazinyl ester	758,506	NF
**Amide**	21.49	9-Octadecenamide, (Z)-	6,551,691	Anti-inflammatory and antibacterial activities
**Alkaloids**	8.31	Indole	2,083,582	Antiproliferative against human tumor cells, Antibacterial, antifungal, and anti-coccidial activities
	9.64	Indole, 3-methyl-	252,685	Antimicrobial, antioxidant, antiviral, anti-HIV, antimalarial, and antituberculosis activities
	12.01	5-Acetyl-2-methylpyridine	137,756	NF
	12.45	2-Acetylpyrido [3,4-d]imidazole	178,259	Anti-inflammatory, anticancer, antimicrobial analgesic, and anti-tubercular activities
	18.38	Naphtho [2,1-d] imidazole	537,389	Anti-inflammatory, anticancer, antimicrobial analgesic, and anti-tubercular activities
	21.96	Dihydroergotamine	27,040,433	Antimigraine activity
	24.66	Benzeneethanamine, 2-fluoro-ß,3,4-trihydroxy-N-isopropyl-	115,115	Anti-inflammatory, antioxidant, and antibacterial activities
**Amine**	4.76	1-Butanamine, 3-methyl-N-(3-methylbutylidene)-	466,907	Antimicrobial activity
	6.09	Benzeneethanamine, N-[(pentafluorophenyl)methylene]-ß,4-bis [(trimethylsilyl)oxy]-	179,683	Anti-inflammatory, antioxidant, and antibacterial activities
	10.14	Benzeneethanamine, N-(3-methylbutylidene)-	1,177,101	Anti-inflammatory, antioxidant, and antibacterial activities
	15.48	Hexadecanamide	106,249	Anti-seborrheic activity
	18.03	2,5-Piperazinedione, 3,6-bis(2-methylpropyl)-	852,049	Antibiotic glycopeptide, anti-ischemic, and anti-dyskinetic activities
	20.97	2,5-Piperazinedione, 3-benzyl-6-isopropyl-	1,546,959	Anti-nematode activity
	27.45	Benzeneethanamine, N-[(3,4-dimethoxyphenyl) methyl]-3,4-dimethoxy-	136,196	Anti-inflammatory, antioxidant, and antibacterial activities
**Ketones**	6.78	2-Piperidinone	6,779,522	Anti-cancer activity
	18.35	4(3H)-Pyrimidinone, 3-ethyl-2,6-dimethyl-	1,195,217	Antitumor activity
**Phenols**	8.28	Phenol, 4-(1,1-dimethylethyl)	1,487,788	Anti-inflammatory and antioxidant activities
	10.43	Tyrosol	7,442,803	Antioxidant, anti-inflammatory, and anticancer activities
	11.72	Butylated hydroxytoluene	80,501	Antioxidant activity
	11.76	2,4-Di-tert-butylphenol	666,258	Antioxidant and antifungal activities
	20.53	Tyrosol, acetate	2,739,903	NF
	22.00	Phenol, 2,4-bis(1-phenylethyl)-	28,300	NF
**Vitamins**	27.17	DL-alpha-Tocopherol acetate	1,057,973	Antidermatitic, antileukemic, antitumor, anti-aging, analgesic, antidiabetic, anti-inflammatory, and antioxidant activities
**Others**	8.50	Naphtho [2,3-b] furan-2-one, 3-[[(benzo [1,3]dioxol-5-ylmethyl)amino]methyl]-8a-methyl-5-methylene-decahydro-	104,639	Anticancer, antimicrobial, anti-inflammatory, analgesic, anthelmintic, diuretic, and antipyretic activities
	13.56	Triisobutyl (3-phenylpropoxy) silane	166,198	Antineoplastic activity
	14.62	Dotriacontyl isobutyl ether	459,568	NF
	23.01	Triphenylphosphine oxide	93,460	Antiarrhythmic and antineoplastic activities

NF, not found; RT, retention time.

A total of eight amino acid metabolites were detected from *B. paranthracis* strain MHSD3. Amino acids and their derivatives regulate the metabolism of carbohydrates and lipids and further produce metabolites (Indira et al., 2019). Fermentation of amino acids such as proline, leucine, and phenylalanine by probiotic microorganisms in the gut results in the production of polyamines, indole, and phenolic compounds that can sustain energy balance and contribute antioxidant and anti-inflammatory properties (Dai et al., 2014; Chugh and Kamal-Eldin, 2020). Phenolic compounds such as tyrosol, phenol, 4-(1,1-dimethylethyl), butylated hydroxytoluene, and 2,4-di-tert-butylphenol were identified in the crude extract, and they have shown a diverse biological activity such as antimicrobial, antioxidant, anticancer, and anti-tumor (Belmonte-Reche et al., 2016; Karkovic Markovic et al., 2019; Ren et al., 2019). Some of the bioactive compounds identified from *B. paranthracis* MHSD3, such as phosphoric acid, sebacic acid, propanoic acid, hexadecane, and 9-octadecenamide, were also identified in the other probiotic strains such as *L. plantarum* DB-2, *L. fermentum* J-1, *Pediococcus acidilactici* M-3, *L. plantarum* SK-3, and *Pediococcus pentosaceus* SM-2 (Yehye et al., 2012). Probiotics produce metabolites that have antimicrobial, antimutagenic, and anticarcinogenic activities. These properties can help eliminate gastrointestinal-related diseases (Bedada et al., 2020; Chaudhary et al., 2020). *B. paranthracis* strain MHSD3 has shown to be rich in a variety of secondary metabolites, especially antibacterial and antimicrobial compounds. The secondary metabolites results are congruent with the functional annotation results; the identification of these compounds showed that *B. paranthracis* MHSD3 is versatile, as it was originally isolated as an endophyte but also possesses probiotic properties.

NRPS and PKS (polyketide synthase) gene clusters are responsible for the production of secondary metabolites with enormous biological activities (Doroghazi and Metcalf, 2013; Khater et al., 2016; Passari et al., 2017). Zheng et al. (2019) and Passari et al. (2017) reported a correlation between the discovery of NRPS/PKS genes and antimicrobial activity of crude extracts against pathogenic strains. In this study, although PKS gene clusters were not detected, NRPS gene clusters were identified, and strain MHSD3 has been shown to secrete an array of secondary metabolites, including antimicrobial compounds, which were effective against pathogenic strains. Further studies are essential to link the secreted secondary metabolites and the *in silico* identified biosynthetic gene clusters and evaluate more of their biotechnological applications.

## Conclusion

The genome analysis of *B*. *paranthracis* strain MHSD3 indicates that the strain is an excellent potential probiotic. *B. paranthracis* strain MHSD3 is a prospective probiotic, and its *in vitro* probiotic assays correspond with the *in silico* probiotic analysis. Strain MHSD3 codes for genes that play a role in acid and bile salt tolerance, adhesion, and antimicrobial activity, which were displayed by survival in a low pH environment, EPS production, and production of secondary metabolites with antimicrobial activity, as well as coding for bacteriocins genes, respectively. Furthermore, strain MHSD3 is susceptible to various antibiotics. The bacteriocins such as sonorensin may help expand their further application in biotechnology, and we recommend the characterization of its bacteriocins. The *in silico* probiotic analysis and *in vitro* probiotic assays indicate strain MHSD3 to be a potential probiotic, and we thus recommend *in vivo* probiotic studies using suitable hosts.

## Data Availability

The data from this Whole Genome Shotgun project has been deposited at DDBJ/ENA/GenBank under the accession JABGBK000000000, BioSample accession number SAMN14911282, and BioProject accession number PRJNA632519. The version described in this paper is JABGBK010000000.
