# Supplementary material for: Genome In Silico and In Vitro Analysis of the Probiotic Properties of a Bacterial Endophyte, Bacillus Paranthracis Strain MHSD3
Source: Front Genet. 2021 Nov 11;12:672149. doi: 10.3389/fgene.2021.672149 (PMC8631869; doi:10.3389/fgene.2021.672149)
Supplement: Supplementary file 1 [file DataSheet1.docx]

**Supplementary Material**

**Table S1: Genome characteristics of *Bacillus paranthracis* MHSD3**

| **Genome characteristic** | **Value** |
| --- | --- |
| Genome size (bp) | 5 396 337 |
| Genomic DNA G+C content (%) | 35.3 |
| Total number of genes | 5 547 |
| Protein coding genes | 5 348 |
| Number of RNAs | 96 |
| rRNA genes | 2, 1, 1 (5S, 16S, 23S) |
| tRNA gene | 87 |
| ncRNAs | 5 |
| Pseudo genes | 103 |

**Table S2: Pairwise dDDH and ANI values between *Bacillus paranthracis* MHSD3 and the *B. cereus* group strains**

| **Type strain** | **dDDH value** | **ANI Value** |
| --- | --- | --- |
| *B. paranthracis* Mn5^T^ | 79.0 | 97.63 |
| *B. pacificus* EB422^T^ | 66.0 | 95.93 |
| *B. tropicus* N24^T^ | 62.7 | 95.53 |
| *B. anthracis* ATCC 14578^T^ | 60.4 | 95.19 |
| *B. fungorum* 17-SMS-01^T^ | 53.0 | 93.59 |
| *B. albus* N35-10-2^T^ | 52.0 | 93.40 |
| *B. wiedmannii* FSL W8-0169^T^ | 51.0 | 93.33 |
| *B. mobilis* 0711P9-1^T^ | 50.6 | 93.19 |
| *B. cereus* ATCC 14579^T^ | 45.1 | 91.76 |
| *B. luti* TD41^T^ | 44.4 | 91.62 |
| *B. thuringiensis* ATCC 10792^T^ | 44.1 | 91.64 |
| *B. toyonensis* BCT-7112^T^ | 42.6 | 91.04 |
| *B. nitratireducens* 4049^T^ | 39.4 | 90.05 |
| *B. proteolyticus* TD42^T^ | 39.2 | 89.88 |
| *B. mycoides* DSM 2048^T^ | 37.9 | 89.47 |
| *B. weihenstephanensis* DSM 11821^T^ | 37.9 | 89.46 |
| *B. paramycoides* NH24A2^T^ | 36.7 | 88.83 |
| *B. pseudomycoides* DSM 12442^T^ | 26.7 | 82.08 |
| *B. cytotoxicus* NVH 391-98^T^ | 25.4 | 81.27 |

**Table S3: Putative genes involved in endophytic lifestyle**

| **Function** | **Genes** | **Gene product** |
| --- | --- | --- |
| **Transcriptional regulator** | *AraC*  *AsnC*  *LrgB*  *LysR* | AraC family transcriptional regulator  Lrp/AsnC family transcriptional regulator  LrgB family protein  LysR family transcriptional regulator |
| **Transporter** | *Lyse*  *MFS*  *ABC*  *NAD* | Lysine exporter protein lyse/ygga  Major facilitator superfamily  Amino acid ABC transporter substrate binding ABC transporter related  NAD(P)-dependent oxidoreductase |
| **Secretion and delivery system** | *-*  *-* | RND family efflux transporter MFP subunit  Type II secretion system F family protein  Type III secretion system protein FlhB, FliP |
| **Plant polymer degradation/modification** | *-*  *-* | Cupin  Alpha/beta hydrolase |
| **Detoxification** | *-*  *-* | Peptidase M4 family protein  SDR family oxidoreductase  2-dehydropantoate 2-reductase |
| **Redox** **potential** **maintenance** | *phbB*  *-* | Acetoacetyl-CoA reductase  Aldehyde dehydrogenase |
| **Others** | *-*  *-*  *-* | Hypothetical protein  2-isopropylmalate synthase  Diaminopimelate decarboxylase |

**Table S4: Cell auto-aggregation and cell hydrophobicity of *Bacillus paranthracis* MHSD3**

|  | **Cell hydrophobicity** | | |  |
| --- | --- | --- | --- | --- |
| **Strain** | **Hexadecane** | **Chloroform** | **Ethyl acetate** | **Auto-aggregation** |
| **MHSD3** | 29 ± 0.69 | 54.28 ± 3.53 | 20.18 ± 0.69 | 78.91 ± 0.00 |

Values are means of triplicate measurements with ± standard deviation

**Table S5: Antibiotic susceptibility of *Bacillus paranthracis* MHSD3**

| **Antibiotic strains** | **Concentration (µg/disc)** | **Zone of inhibition (mm)** | **Resistance or Sensitive** |
| --- | --- | --- | --- |
| Erythromycin | 15 | 24 | S |
| Gentamicin | 10 | 19 | I |
| Metronidazole | 5 | 0 | R |
| Polymyxin B | 300 | 9 | R |
| Cefuroxime | 30 | 19 | I |
| Cefalexin | 30 | 26 | S |
| Ciprofloxacin | 5 | 24 | S |

R= resistant (≤ 14 mm); I = Intermediate (15-19 mm); S= Sensitive (≥ 20 mm)

**Table S6: Minimum inhibition concentration of *Bacillus paranthracis* MHSD3 crude extract**

| **Pathogenic strains** | **Concentration (mg/mL)** |
| --- | --- |
| *Pseudomonas aeruginosa* | 15 |
| *Klebsiella oxytoca* | 15 |
| *Klebsiella pneumonia* | 3.75 |
| *Escherichia coli* | 15 |
| *Staphylococcus epidermis* | 1.87 |
| *Veillonella purvula* | 3.75 |
| *Staphylococcus auerus* | 7.5 |
| *Enterococcus faecium* | 15 |
| *Bacillus cereus* | - |
| *Staphylococcus saprophyticus* | 1.87 |
| *Mycobacterium smegmatis* |  |

- : No inhibition; values are statistically significant at p ≤0.05


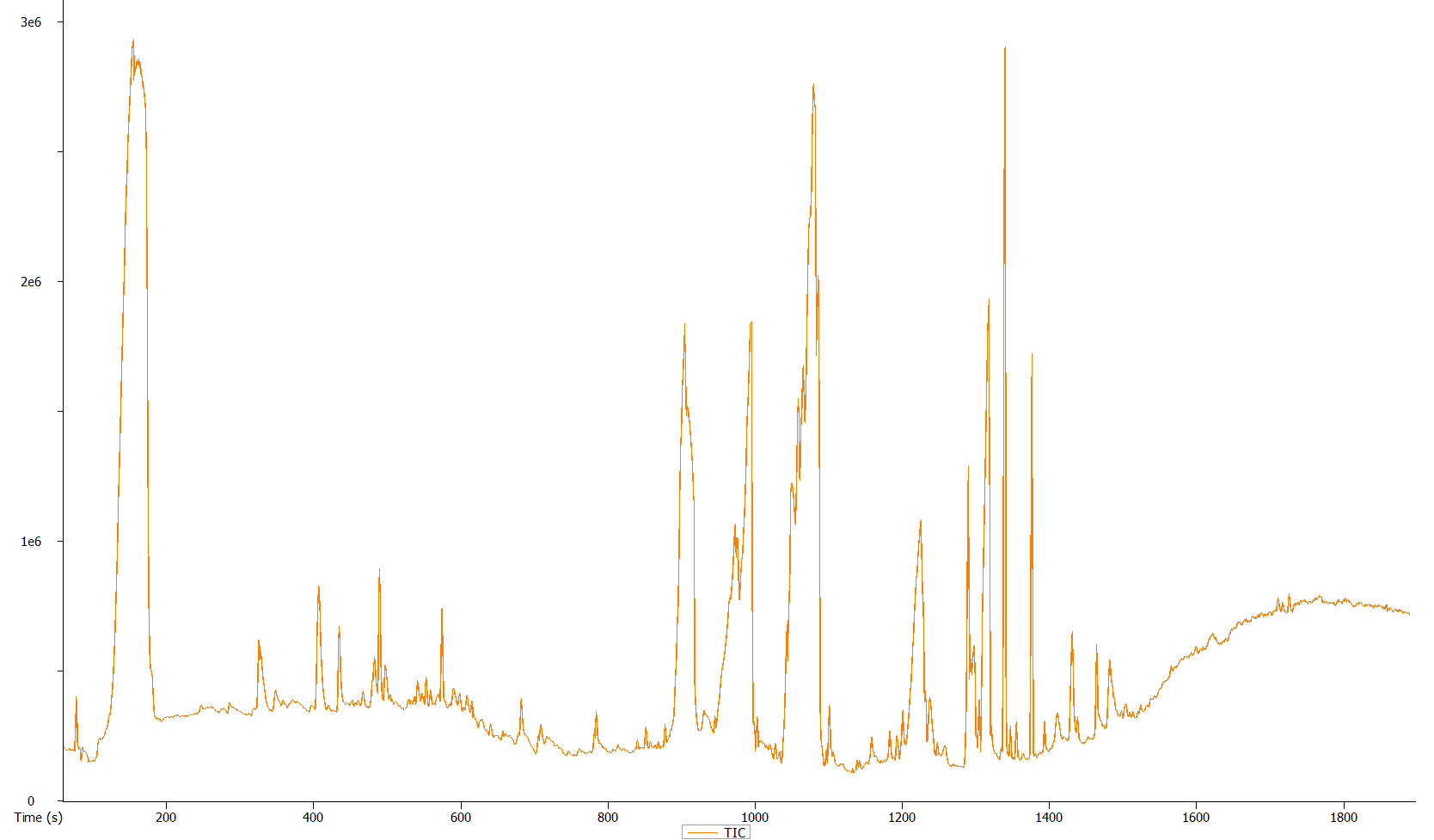


**FIGURE S1**. GC-MS chromatogram of *Bacillus paranthracis* strain MHSD3’s crude extract.
